# The protective activity of genistein against bone and cartilage diseases

**DOI:** 10.3389/fphar.2022.1016981

**Published:** 2022-09-08

**Authors:** Zhenyu Wu, Luying Liu

**Affiliations:** ^1^ First Affiliated Hospital of Gannan Medical University, Ganzhou, China; ^2^ First Clinical Medical College of Gannan Medical University, Ganzhou, China

**Keywords:** genistein, osteoporosis, osteoarthritis, rheumatoid arthritis, intervertebral disc degeneration

## Abstract

Genistein, a natural isoflavone rich in soybean and leguminous plants, has been shown various biological effects, such as anti-inflammation, anti-oxidation, anti-cancer, and bone/cartilage protection. Due to the structural similarity to estrogen, genistein exhibits estrogen-like activity in protecting against osteoporosis and osteoarthritis. Furthermore, genistein has been considered as an inhibitor of tyrosine kinase, which has been found to be dysregulated in the pathological development of osteoporosis, osteoarthritis, and intervertebral disc degeneration (IDD). Many signaling pathways, such as MAPK, NF-κB, and NRF2/HO-1, are involved in the regulatory activity of genistein in protecting against bone and cartilage diseases. The potential molecular mechanisms of genistein in therapeutic management of bone and cartilage diseases have been investigated, but remain to be fully understood. In this article, we mainly discuss the current knowledge of genistein in protecting against bone and cartilage diseases, such as osteoporosis, osteoarthritis, rheumatoid arthritis (RA), and IDD.

## Introduction

**FIGURE 1:**
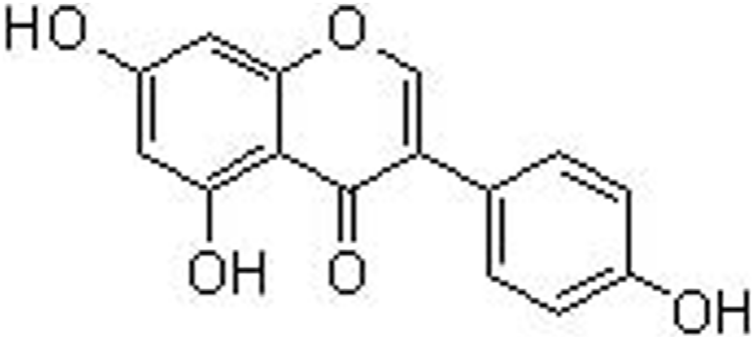
The chemical structure of genistein.

Today, the growing interest in functional foods have attracted strong attention, due to its contribution to health by providing basic nutrition and biological activities against diseases. For example, soybean is the richest source of isoflavone genistein (a molecular formula of C_15_H_10_O_5_, a molecular lweight of 270 g/mol, and chemical name as 4’,5,7-trihydroxyisoflavone, Figure 1), constituting 5.6–276 mg/100 g (Yu et al., 2021). Genistein is also a secondary metabolite and often found in leguminous plants, seeds, fruits, and vegetables with a concentration of 0.2–0.6 mg/100 g (Liggins et al., 2000). However, most of isoflavones, including genistein, presented in the natural sources are in the forms of glycosylation, and they can turn into aglycones by the food processing (Smeriglio et al., 2019). Genistein can be one of the classic isoflavones with phytoestrogen activity by structurally or functionally mimicking mammalian estrogen 17β-estradiol (Barnes et al., 2000). Other subtypes of natural occurring phytoestrogens involve lignans, flavones, coumestans, chalcones, and prenylflavonoids. These compounds possess a diphenolic structure, providing a structural basis for synthesizing the potential diethylstilbestrol and hexestrol (Suen et al., 2022). Interestingly, genistein exhibits a critical role in various biological and pharmacological actions, including anti-oxidation, anti-inflammation, anti-cancer, anti-diabetes, neuroprotection, liver protection, and bone protection (Sharifi-Rad et al., 2021). Many signaling cascades and networks, such as estrogen receptor (ERα and ERβ) pathway, Wnt/β-catenin, PI3K/AKT, NF-κB, and MAPK are involved in the biological functions regulated by genistein (Mobeen et al., 2022).

Genistein has been reported to suppress hypoxia-induced ROS generation and ameliorate oxidative stress-induced cognitive impairment in mice hippocampus (Rumman et al., 2021). In addition, genistein could target to inhibit the activity of Keap1 and indirectly up regulate the expression of NRF2, promoting anti-oxidative stress and protecting against neurodegenerative diseases (Xi et al., 2022). Flavonoids and genistein exhibit comprehensive anti-inflammatory activity, which has been sophisticated reviewed (Al-Khayri et al., 2022; Goh et al., 2022). The classical MAPK/NF-κB signaling pathway has been shown to be suppressed by genistein in lipopolysaccharide (LPS)-induced RAW264.7 cells (Jia et al., 2020). Similarly, the anti-cancer activity of genistein has been also comprehensively reviewed (Hou, 2022). Genistein exhibits cytotoxicity to many cancers, such as breast cancer (Pawlicka and Filip, 2022), prostate cancer (Ji et al., 2022), liver cancer (Zhang et al., 2019), and kidney cancer (Imai-Sumida et al., 2020). In mammalians, isoflavones, such as genistein, can exhibit estrogen-like biological functions, due to the structural similarity between genistein and estrogen. Particularly, genistein can be considered as an estrogen agonist to exhibit a synergistic action with endogenous hormones, or as an estrogen antagonist to block the biological effects of estrogen receptors (ERα and ERβ) (Zaheer and Humayoun Akhtar, 2017). However, unlike estrogen, genistein prefers to interact with ERβ (a relative binding affinity of 87% of 17β-estradiol) over ERα (4% of 17β-estradiol) in a solid-phase competitive experiment (Kuiper et al., 1998). Interestingly, estrogen play a critical role in the homeostasis of musculoskeletal system (Essex et al., 2022). In this article, we mainly discuss the potential roles of genistein in the protection against bone and cartilage diseases.

## The protective activity of genistein against bone and cartilage diseases

### Genistein and osteoporosis

Osteoporosis is featured by a loss of bone mass. In the United States, about 55% of the persons with age of more than 50 have osteoporosis or low bone density, which may result in fractures. By 2025, more than three million/year fractures induced by osteoporosis will be expected, accompanied by more than $25 billion/year health cost (Burge et al., 2007). A positive relationship between soybean isoflavone intake and bone mineral density (BMD) has been observed in the postmenopausal women, which indicates that isoflavone may, at least partially, restore the metabolic balance between bone formation and resorption (Somekawa et al., 2001). Soybean isoflavones have been demonstrated the beneficial effects of estrogen-like on bone without producing obvious untoward effects as natural estrogen, such as hyperplasia, increased risk of breast cancers, and cardiovascular diseases (Setchell, 2001).

The high dietary phytoestrogen consumption has been demonstrated with increased BMD by cross-sectional analysis in postmenopausal but not premenopausal Chinese women (Mei et al., 2001). Consistently, in ovariectomized (OVX) rats, genistein (oral administration at the dose of 10 mg/kg for 12 weeks) has been reported to exhibit a stimulatory effect on bone formation and an inhibitory activity against bone resorption (Hertrampf et al., 2009) (Table 1). In OVX-induced rat osteoporosis, soybean genistein at the dose of 4.5 mg/kg or 9 mg/kg exhibits protective activity against osteoporosis after 4- week treatment. In contrast, genistein at the dose of 18 mg/kg shows less beneficial effects on bone loss. This might be associated with the estrogen antagonist activity of genistein (Wang et al., 2006). The protective of genistein against osteoporosis has been also demonstrated in orchidectomized rats by improving the trabecular micro-architecture of proximal tibia, increasing histomorphometrical changes in thyroid glands, and lowing the levels of circulating thyroid hormone (Filipović et al., 2018).

**TABLE 1:** The biological effects of genistein on osteoporosis.

Models/doses	Main results	Conclusion	Ref
postmenopausal women/54 mg daily for 24 months	Increase BMD and enhance the levels of ALP and IGF-1	Genistein has positive effects on BMD	Marini et al. (2007)
postmenopausal women/54 mg daily for 6/12 months	Increase the serum levels of the bone specific ALP and osteocalcin; increase BMD in the femur and lumbar spine	Genistein has positive effects against bone loss	Morabito et al. (2002)
OVX rats/42 mg/kg for 12 weeks	Does not affect uterine wet weights; increase trabecular BMD and serum Pyd; reduce OPN	Genistein shows bone protection without stimulation of uterine wet weight	Hertrampf et al. (2009)
OVX rats/4.5 or 9 mg/kg for 12 weeks	Increase BMD in the femur and tibia, enhance the contents of Ca, Mg, and P, and improve bone histology and morphology	Genistein prevents bone loss	Wang et al. (2006)
Rat osteoblasts/100 μM	Increase ALP, RUNX2, and OCN genes expression in ERα-dependent manner	Genistein protects bone via triggering ERα-mediated osteogenesis-associated gene expressions	Wu et al. (2020)
RAW264.7/0.1–100 μM	Synergy with alendronate to suppress RANKL-induced osteoclast differentiation	Bisphosphonate and genistein combination prevent bone resorption	Yamaguchi and Levy, (2017)
RAW264.7/1–20 μM	Suppress the expression of NOX-1 and mitochondrial dysfunctions, stimulate NRF2/HO-1 signaling	Genistein exhibits anti-oxidative stress and inhibits RNAKL-induced osteoclast differentiation	Lee et al. (2014)
Rat osteoclast/10^−7^–10^−5^ M	The protein kinase activity is decreased	Genistein suppress osteoclast functions by inhibiting the activity of protein kinase	Gao and Yamaguchi, (2000)
Rat with GIOP/5 mg/kg i.p. for 60 days	Increase b-ALP and OPG contents and reduce CTX level	Genistein prevents against GIOP	Bitto et al. (2009b)
T2DM rats/10 and 30 mg/kg for 8 weeks	Lower fasting blood glucose, decrease the productions of IL-6, TNFα, RANKL, and PPARγ, and increase the expression of OPG, RUNX2, and β-catenin	Genistein improves abnormal bone metabolism in STZ-induced T2DM rats	Lu et al. (2020)
MTX-treated rats/20 mg/kg for 5 days	Preserve body weight gain, inhibit osteoclast formation	Genistein suppresses MTX-induced osteoclatogenesis	King et al. (2015)
OVX rats/5 mg/kg for 10 days	Recover bone structure, histomorphometric parameters, and OPG/RANKL ratio	Genistein synergy with silicon to protect against osteoporosis	Chen et al. (2019)

It has been known that genistein shows much higher affinity to ERβ over ERα. However, Genistein can stimulates the expression of both ERα and ERβ and promote the proliferative activity in MC3T3-E1 cells (Ye et al., 2018). In bone marrow stromal progenitor cells (BMSCs), genistein induces ERα/ERβ-initiated differentiation and maturation of BMSCs by increasing the activity of ER, p38MAPK-RUNX2, and NO/cGMP signaling pathways and decreasing the formation of osteoclasts and the resorption of bone metabolism by blocking the activity of NF-κB signaling (Ming et al., 2013). Recently, genistein increase the activity of alkaline phosphatase (ALP) time-dependently and the expression of osteogenesis-related osteocalcin and RUNX2 in rat osteoblasts by up regulating the expression of ERα (Wu et al., 2020) (Table 1). Consistently, it is fascinated that genistein promotes the differentiation-associated genes expression and osteoblast mineralization by increasing the expression of ERα and activating the activity of MAPK/NF-κB/AP-1 signaling pathway in MC3T3-E1 cells (Liao et al., 2014).

On the other side, it has been reported that the effects of estrogen on osteoblast are associated with the phages of differentiation and the isoforms expression of ER. The ratio of ERβ/ERα increases in women with sex steroids deficiency (Ireland et al., 2002). In ovariectomized (OVX) rats, genistein (5 mg/kg/day) may effectively preserved the biomechanical quality of the trabecular bone. However, it does not prevent the microstructural degeneration or improve the bone mineral density. The explanation might be associated with the differentiated expression between ERα and ERβ (Dai et al., 2008). Another study also exhibits that oral isoflavone administration has no effects on the bone loss in oophorectomized rats (Draper et al., 1997). In OVX monkeys, dietary soy phytoestrogens be also poor in protecting against bone loss resulting from the deficiency of estrogen (Register et al., 2003). The discrepancy might be related to the specifical situations of *in vivo* and *in vitro* models, and more efforts are still needed for clear elucidation.

**FIGURE 2:**
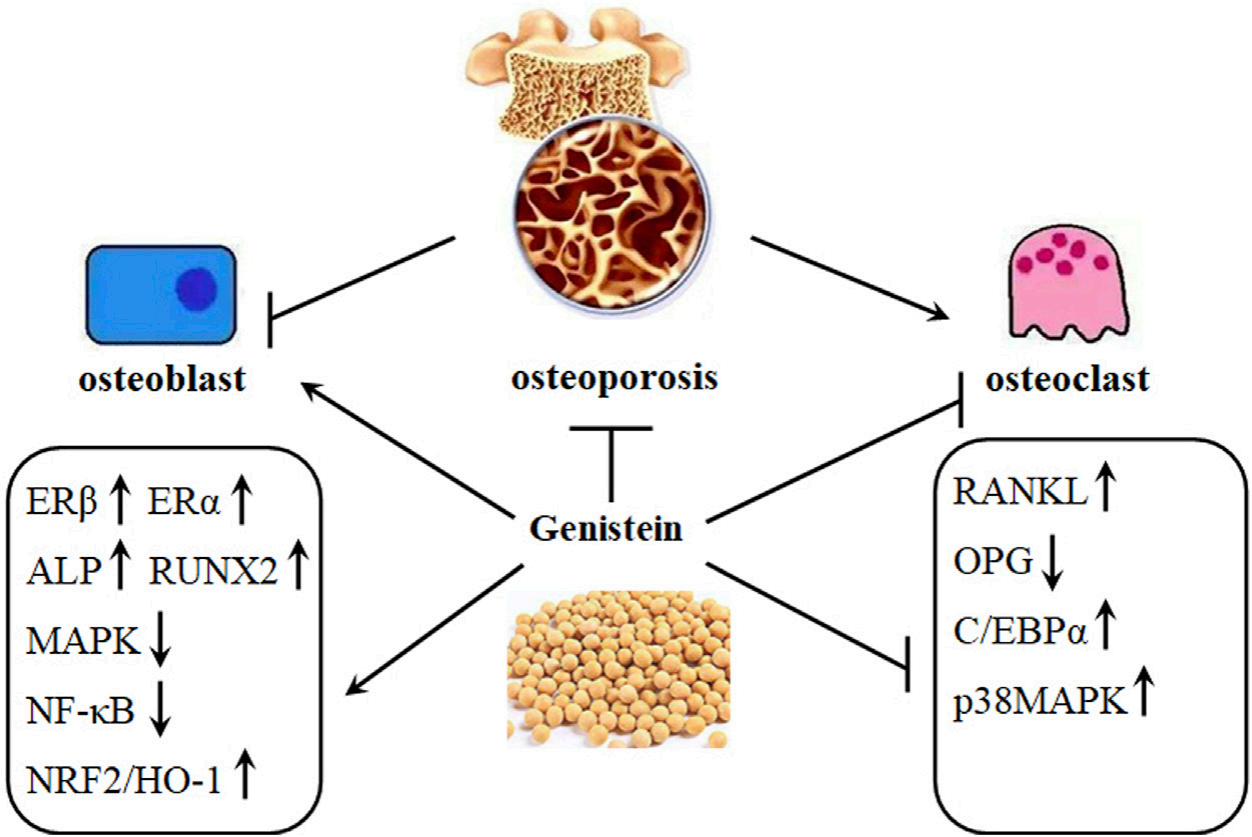
Genistein protects against osteoporosis development. Osteoporosis is characterized by decreased activity of osteoblasts and increased activity of osteoclasts. This imbalance can be restored by genistein, as shown by increased expression of ERβ, ERα, ALP, RUNX2, and NRF2/HO-1 and decreased expression of MAPK, NF-κB, C/EBPα, and RANKL/OPG ratio.

Bone, a dynamic tissue, constantly undergoes remodeling by osteoclast-mediated resorption and osteoblast-regulated formation (Figure 2). Bone mass deterioration with aging may result in osteoporosis. Genistein positively regulates bone cell metabolism by potentiating bone turnover towards bone formation, implying stimulation of osteoblast activity and inhibition of osteoclast functions (Bitto et al., 2010). In pre-osteoclastic RAW 264.7 cells, genistein can decrease RANKL-induced osteoclastic differentiation *in vitro*. Furthermore, genistein may synergize with bisphosphonate alendronate to inhibit the osteoclastic differentiation induced by RANKL, which providing a novel strategy for clinic prevention and treatment of osteoporosis (Yamaguchi and Levy, 2017) (Table 1). Genistein also limits the generation of ROS by up regulation of NRF2/HO-1 signaling pathway, down regulation of NADPH oxidase 1 (NOX1), and inhibition of disrupting the mitochondrial electron transport chain system in RANKL-treated RAW264.7 cells (Lee et al., 2014). Another study reports that the inhibitory effects of genistein on dibutyryl cyclic adenosine monophosphate (cAMP)-induced osteoclast-like cell formation in mouse marrow culture (Gao and Yamaguchi, 1999a). The inhibitory effects of genistein on rat osteoclasts might be associated with inactivation of protein kinase and activation of protein tyrosine phosphatase (Gao and Yamaguchi, 2000). Another molecular mechanism involved in the inhibitory effects against osteoclasts formation might be associated with the increasing entry of calcium into osteoclasts by genistein, which finally induces osteoclast apoptosis. This inhibition is not affected by tamoxifen, which suggests that the inhibitory activity against osteoclast is not related to estrogen (Gao and Yamaguchi, 1999b). However, this effects of increased intracellular calcium by genistein may be regulated by inhibiting inward-rectifier K+ channel current, regardless of the activity of genistein on tyrosine kinase (Okamoto et al., 2001).

The wide use of glucocorticoids for treating inflammatory responses may cause a secondary osteoporosis, e.g., glucocorticoid-induced osteoporosis (GIOP). It has been accepted that glucocorticoids can decrease bone mass and then increase fracture risks. Alternative therapeutic approaches have been reached, and genistein has been shown to reverse GIOP in rats (Bitto et al., 2009a). Consistently, genistein protects against GIOP in rats, as indicated by increased BMD, b-ALP, and OPG and decreased carboxy-terminal collagen crosslink (CTX) (Bitto et al., 2009b) (Table 1). Both osteoblasts and adipocytes are differentiated from BMSCs. Glucocorticoid-induced lipid accumulation has been demonstrated to be negative to bone mass (Zheng et al., 2021). It is evident that bone loss increases with ageing. Meanwhile, the activity of osteoblastogenesis decreased, accompanied by enhanced adipogenesis (Yang et al., 2021). Consistently, genistein has been reported to increase the differentiation of osteoblasts and decrease the differentiation of adipocytes in human BMSCs by suppressing the activity of CCAAT/enhancer-binding proteins (C/EBPs) and peroxisome proliferator-activated receptor γ (PPARγ) (Yu et al., 2012). In addition, type II diabetes also induce impairment of bone microstructure, loss of BMD, and increased number of adipocytes and osteoclasts in rats, and genistein (30 mg/kg) might ameliorate the pathological changes in streptozotocin (STZ)-induced diabetic rats by decreasing the expression of RANKL and PPARγ and increasing the expression of OPG, RUNX-2, and β-catenin (Lu et al., 2020).

Postmenopausal osteoporosis has been shown to be related to the balance of OPG and RANKL system, which has become a potential pharmacological target for osteoporosis treatment (Figure 2). Decreased OPG/RANKL expression is found *in vitro*, and genistein can selectively ameliorate parathyroid hormone (PTH)-induced catabolic effects in bone by increasing the expression of OPG/RANKL system (Chen and Wong, 2006). Consistently, genistein treatment is associated with OPG/RANKL system and BMD in OVX rats (Bitto et al., 2008). Interestingly, genistein also protects against methotrexate (MTX)-induced bone loss, as shown by decreased osteoclast formation and down regulated C/EBPα expression. However, administration of genistein (20 mg/kg) does not affect MTX-induced damages in bone volume, trabecular architecture, and metaphyseal mRNA expression of pro-osteoclastogenic cytokines in rats (King et al., 2015). Zinc, an essential trace element included in soybeans, has been reported to stimulate bone formation and inhibit bone resorption, enhancing bone mass. The beneficial effects of zinc on bone metabolism have been discussed. Genistein has been demonstrated to synergize with zinc to protect against osteoporosis (Yamaguchi, 2012). Genistein has been also demonstrated the synergistic effects with silicon against OVX-induced BMD decrease and bone loss in rats, as shown that genistein can significantly reduce the expression of RANKL and enhance the levels of OPG in the serum and bone tissues (Chen et al., 2019).

BMD is a useful tool for diagnosing osteoporosis and predicting the risk of fracture. Particularly for the latter, BMD is not comprehensive but limited. Alternatively, bone turnover markers (BTMs) have been demonstrated to be important in bone mass, height, and other growth factors (Léger et al., 2007). BTMs involves several enzymes that originate from bone cells and bone matrix components. The factors in BTMs associating with osteoblasts activity are serum osteocalcin (OC) in form of N-terminal midmolecule fragment (N-MID) and undercarboxylated osteocalcin (UcOC), N-terminal procollagen of type I collagen (P1NP), and C-terminal procollagen of type I collagen (P1CP). In contrast, factors relating to osteoclast activity are cross-linked N-telopeptide of type I collagen (NTX), cross-linked C-telopeptide of type I collagen (CTX), tartrate-resistant acid phosphatase (TRAP), and pyridinoline (Pyd). During the degradation of mature collagen, the C-terminal peptide α aspartic acid is transformed to β aspartic acid. Thus, β-CTX can be used to reflect the status of bone resorption (Hu et al., 2013). At the dose of 54 mg/kg for 6 weeks, genistein can significantly increase the generation of b-ALP and decrease the production of CTX in OVX rats (Bitto et al., 2011). Interestingly, genistein at the dose of 10 mg/kg for 12 weeks in OVX rats also obtains similar therapeutic results (Bitto et al., 2008). Another study shows that genistein enhances the expression of ALP, OC, Col1, and RUNX2 and decreases the production of TRAP-5b in OVX rats (Song et al., 2014). However, other study reports that genistein can significantly lower the production of CTX and RANKL, promoting bone formation and suppressing bone resorption. However, these markers do not reach statistically difference (Qi, 2018). In a clinical trial, genistein is reported to show no effects on bone remodeling and no additional effects on the expression of P1NP and CTX by combination with calcium and vitamin D (Pérez-Alonso et al., 2017). Thus, more efforts are still needed for further elucidation.

Collectively, several animal models induced by OVX, glucocorticoids, or MTX have been used to demonstrated the protective activity of genistein with a possible mechanism of activation of ERβ/ERα and NRF2/HO-1 pathways and inhibition of RANKL/NF-κB pathways. In clinical study, genistein protects against osteoporosis by promoting bone formation and suppressing bone resorption.

### Genistein and osteoarthritis

**FIGURE 3:**
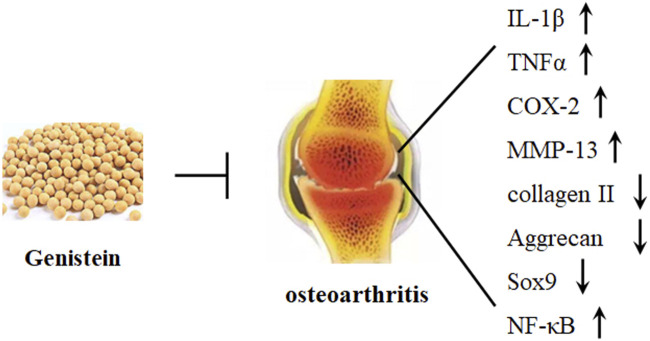
Genistein protects against osteoarthritis development. Genistein can significantly decrease the expression of IL-1β, TNFα, COX-2, MMP-13, and NF-κB and increase the expression of Sox9, collagen II, and aggrecan in chondrocytes.

Osteoarthritis (OA), a chronic degenerative joint disease, is characterized by cartilage matrix structural degradation and chondrocyte degeneration. The main pathological changes of OA are associated with excessive chondrocyte apoptosis, abnormal chondrocyte metabolism, and progressive articular cartilage degeneration, which leading to joint pain and disability (Guilak et al., 2018). Various factors, such as injury, genetic mutation, ageing, and excessive weight, have been involved in the etiological study of OA (Loeser et al., 2012). Currently, no effective therapeutic approaches are available to cure OA, and treatment is only limited to symptom relief. Mechanically, the imbalance between anabolism and catabolism promotes various biochemical and biophysical changes, leading to structural degradation. Cartilage-degrading enzymes, such as MMP-13 and a disintegrin and metalloprotease with thrombospondin motif 4/5 (ADAMTS4/5) have been reported to target ECM degradation and considered as the critical factors in the pathogenesis of OA development (Figure 3). Collagens and proteoglycans (PG) are the major structural molecules in the extracellular matrix (ECM). PG is constituted by low and high sulfated glycosaminoglycans (GAG), and the biosynthesis of GAG is mediated by insulin in the cartilage growth plate. It has been demonstrated that genistein promotes insulin-stimulated sulfate incorporation, benefiting articular cartilage metabolism (Claassen et al., 2008).

Homeostasis of articular cartilage is maintained through modulation of various physiological and pathological activity. Aging has been considered as a major risk factor for OA pathogenesis, and aged chondrocytes are associated with senescent phenotypes (McCulloch et al., 2017). Particularly, senescent chondrocytes, due to dysfunction of autophagy, have been marked by decreased resistance to oxidative stress and imbalanced cellular homeostasis (Kurakazu et al., 2021). Another risk factor is synovitis, which is often observed in the pathological changes of OA joints. Severe synovitis has been associated with cartilage erosion (Xie et al., 2020). Inflammation has been believed to be associated with the development of OA. Several pro-inflammatory cytokines, such as IL-1β, IL-6, and TNFα, participate in the pathophysiological processes of OA metabolism (Shi et al., 2022). Mechanical overloading stress also plays a critical role in promoting chondrocyte senescence and inducing OA pathogenesis. This might be related to reduction of FBXW7 expression and FBXW7-mediated degradation, which result in activation of JNK signaling pathway (Zhang et al., 2022).

Specifically, mechanical overload can induce generation of ROS, oxidative stress, and cartilage degeneration. Piezol, an ion channel, has been reported to modulate mechanosensory transduction, and the transient receptor potential vanilloid 4 (TRPV4) is a Ca^2+^ ion channel. Activation of both piezol and TRPV4 may lead to the influx of Ca^2+^, which can induce mitochondrial dysfunction and ROS accumulation (Davalli et al., 2016). NRF2, a central factor in regulating the expression of antioxidant system, has been reported to interact with NF-κB signaling pathway. Recently, it has been studied that genistein decreases the expression of IL-1β-induced inflammatory cytokines by activating NRF2/HO-1 signaling pathway in human OA chondrocytes. In addition, genistein suppresses IL-1β-induced generation of NOS2, COX-2, and MMP-13, which have been demonstrated to induce chondrocytes apoptosis and the degradation of ECM (Liu et al., 2019) (Table 2). Consistently, genistein also dose-dependently inhibits IL-1β-induced the production of TNFα, the decrease of collagen II and aggrecan, and the apoptosis of chondrocytes by stimulating the expression of ERα *in vivo* and *in vitro*, alleviating the degradation of OA cartilage (Zou et al., 2020) (Figure 3).

**TABLE 2:** The biological effects of genistein on osteoarthritis.

Models/doses	Main results	Conclusions	Ref
Human chondrocytes/10 μM	Inhibit IL-1β-induced NOS2, COX-2, and MMPs and increase NRF2/HO-1 signaling	Genistein acts as an alternative anti-inflammatory agent	Liu et al. (2019)
Human chondrocytes/25–100 μM	Inhibit IL-1β and TNFα expression, increase collagen II and aggrecan, and decrease cell apoptosis	Genistein treats OA by anti-inflammation and anti-apoptosis	Zou et al. (2020)
Human chondrocytes/50 and100 μM	Reduce LPS-induced COX-2 but not COX-1 expression	Genistein inhibits inflammatory responses	Hooshmand et al. (2007)
Rabbit chondrocytes/6–24 μg/ml	Suppress PAP-induced chondrocytes proliferation	Genistein acts as a PTK inhibitor to regulate PAP activity	Lin et al. (2011)
Rat TMJOA/30 and 180 mg/kg for 4 weeks	Decrease the expression of IL-1β and TNFα, inhibit p65 nuclear translocation	Genistein protects against OA by inhibiting NF-κB signaling	Yuan et al. (2019)

The OA prevalence increases in women after menopause. Data from clinical research have demonstrated the relationship between estrogen deficiency and OA development (Gokhale et al., 2004). However, estrogen replacement fails in OA treatment. This suggests that the correlation between OA development and estrogen deficiency is rather complicate (Felson and Nevitt, 1998). Cartilage is a tissue sensitive to estrogen, and both ERα and ERβ are present in chondrocytes, which are the unique cell type in cartilage. In addition, it has been reported that articular chondrocytes have the capacity to synthesize estrogen by themselves, without depending on the external estrogen metabolism. However, the biological effects of estrogen on the metabolism of cartilage and chondrocytes are rather complex that they can be positive or negative (Schicht et al., 2014). A study shows that 17β-estradiol functions to promote chondrocytes proliferation in the growth plate and inhibit spontaneous apoptosis (Chagin et al., 2006). It has been demonstrated that 17β-estradiol exhibits inhibitory activity against MMP-13 expression by up regulating the expression of miR-140 in human articular chondrocytes, leading to protection against IL-1β-induced ECM degradation in cartilage (Liang et al., 2016). Genistein has been reported to decrease the expression of COX-2, NO, and IL-1β in LPS-treated chondrocytes, and this has been suggested to associated with the expression of ERβ (Hooshmand et al., 2007).

ATDC5 cells are often used for investigation of chondrogenic differentiation in endochondral ossification. Recently, genistein (10 μM) can inhibit chondrocyte differentiation in ATDC5 cells by suppressing the expression of Sox9, Col2a1, Acan, and TGFβ1. Furthermore, genistein decreases calcium deposition and mineralization but increase non-chondrogenic mineralization (Kitagawa et al., 2021). Protein kinases, particularly tyrosine kinases, has been shown to regulate cell differentiation and proliferation. Genistein has been considered as an inhibitor of tyrosine kinase, and it can block Pilose antler polypeptides-induced proliferation and differentiation of chondrocytes isolated from the knee cartilages of Zealand white rabbits (Lin et al., 2011) (Table 2).

Temporomandibular joint osteoarthritis (TMJOA) is influenced by inflammatory responses. NF-κB signaling has been considered as the central regulator in the inflammatory responses and immune process. Genistein has been known for anti-inflammatory activity, and it is believed to suppress the activity of NF-κB signaling pathway and its downstream factors, such as IL-1β and TNFα in rat TMJOA (Yuan et al., 2019) (Table 2). The balance of Bcl-2 and Bax controls the processes of apoptosis in cells. Suppression of NF-κB signaling by genistein has been shown to decrease the ratio of Bax/Bcl-2, leading to inhibition of chondrocytes apoptosis and amelioration of OA development (Huang et al., 2004; Yuan et al., 2019).

Collectively, genistein exhibits anti-inflammatory and anti-oxidative activities against OA pathological development. Mechanical overload and estrogen deficiency are also involved in the pathogenesis of OA, and genistein also exhibits protective effects to ameliorate these disorders.

### Genistein and rheumatoid arthritis

**FIGURE 4:**
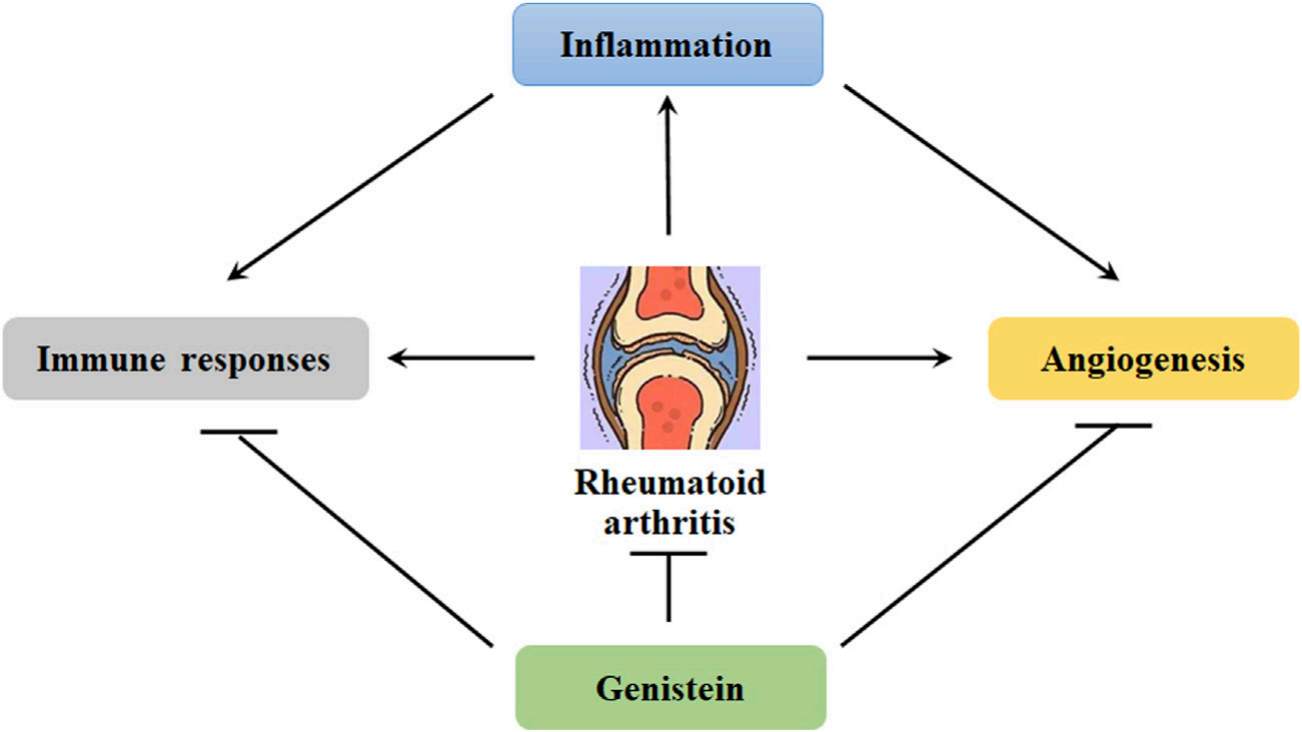
Genistein inhibits the progression of rheumatoid arthritis. Chronic inflammation can induce angiogenesis. In addition, inflammation also induce immune responses, promoting the transformation of fibroblast-like synoviocytes. These abnormalities can be effectively blocked by genistein, protecting against rheumatoid arthritis development.

Rheumatoid arthritis (RA) is defined as a chronic and systemic autoimmune disease, and it is associated with inflammatory disorder, which leads to joint pain, swelling, and stiffness. Clinically, RA is more often found in women than men and diagnosed before the age of 60 (MacGregor et al., 2000). Etiological study shows that the factors causing RA pathogenesis are still unclear. Nowadays, genetic disorder and environment are believed to be the contributors. However, the potential molecular mechanisms that regulating the pathological development of RA still need more efforts. RA is associated with synoviocytes proliferation, inflammatory cells infiltration, and progressive joint erosion (Figure 4). Generally, there are only one or two cell layers in the synovial intimal lining of joints. In the situation of inflammation, the cell layers may increase and become 4–10 layers in the lining. This might be due to the recruitment of other cell types, such as macrophage cells and fibroblast-like cells. In addition, oxidative stress also facilitates the progression of RA. In collagen-induced rat arthritis, genistein inhibits the productions of MDA and restores the activity of paraoxonase and arylesterase (Mohammadshahi et al., 2013) (Table 3). Consistently, a study of 47 RA cases shows that the levels of lipid hydroperoxides (LOOHs) is found to be higher, and the productions of free sulfhydryl and the activity of paraoxonase, arylesterase, and ceruloplasmin are lower (Isik et al., 2007). Genistein has been demonstrated to reduce the generation of lipid peroxidation and exhibit anti-oxidative stress in OVX rats (Witayavanitkul et al., 2020).

**TABLE 3:** The biological effects of genistein on RA and IDD.

Models/doses	Main results	Conclusions	Ref
CIA rats/20 mg/kg for 50 days	Decrease the levels of serum MDA and lipid, restore the activity of paraoxonase and arylesterase	Genistein protects against by exhibiting anti-oxidative activity	Mohammadshahi et al. (2013)
CIA rats/20 mg/kg for 50 days	Reduce ear thickness and the productions of TNFα, IL-6, adiponectin, and leptin	Genistein improve RA symptoms	Mohammad-Shahi et al. (2011)
MH7A/20 μM	Decrease TNFα-induced IL-1β, IL-6, and IL-8 productions, inhibit AKT, IκBα, IKKα/β, and p65 phosphorylation, promoting AMPK	Genistein alleviates RA by inhibiting ROS/AKT/NF-κB and promoting AMPK activity	Li et al. (2014)
RA-FLS/37 μM	Inhibit IL-1β-, TNFα-, or EGF-induced proliferation, 3H-thymidine incorporation, and MMP-9 expression	Genistein suppresses anchoage dependent and independent growth of RA-FLS	Zhang et al. (2012)
CIA mice/5 mg/kg for 10 days	Decrease serum levels of IL-1β, IL-6, and TNFα and the expression of VEGF	Genistein inhibits angiogenesis in the synovial tissue	Hu et al. (2016)
MH7A/10–25 μM	Decrease the expression of IL-6, VEGF, and STAT3	Genistein inhibits IL-6-induced angiogenesis by inhibiting JAK2/STAT3 axis	Cheng et al. (2020)
CIA rats/1 mg/kg for 42 days	Decrease the production of IFNγ and IL-4, maintain Th1/Th2 balance	Genistein inhibits RA-induced inflammation and modulates the immune system	Wang et al. (2008)
CIA mice/20 mg/kg for 63 days	Decrease the activity of NF-κB and NFATc1/c-Fos signaling and increase IgG glycosylation	Genistein alleviate RA by inhibiting inflammation and increasing IgG glycosylation	Du et al. (2020)
NP cells/100 μM	Inhibit TBHP-induced apoptosis and MMPs expression, increase collagen-II production and NRF2 expression	Genistein inhibits NP cells degeneration by activating NRF2 signaling	Wang et al. (2019)
NP cells/10–30 μM IDD rats/10 and 20 μg/ml for 4 weeks	Inhibit p38 phosphorylation, decrease IL-1β and TNFα expression, increase COL2A1 and aggrecan expression	Genistein suppress IDD by inhibiting p38 MAPK signaling	Ge et al. (2020)

Substantial studies on the pathophysiology of RA indicate that activation of inflammatory signaling pathways may result in alternation of immune system and onset of disease (Figure 4). The aberrant inflammatory signaling pathways in RA development are featured as the imbalance between anti-inflammation and pro-inflammation and induced by the alterations in the profile of Th1 cells (Attur et al., 2022). Recently, the biological roles of G-protein-coupled receptor (GPCRs), including chemokine receptors, melanocortin receptors, lipid metabolism-related receptors, adenosine receptors and other inflammation related receptors, have been demonstrated in the pathogenesis of RA in inflammation, lipid metabolism, angiogenesis, and bone destruction (Zhao et al., 2022). The NLRP3 inflammasome can be a critical source of IL-1 and IL-8, promoting the progression of RA. Increased expression of NLRP3 mRNA and NLRP3-related protein in monocytes, macrophages, and dendritic cells have been observed in patients with RA (Guo et al., 2018). The inflammasome can be activated in non-phagocytes, such as T cells and epithelial cells. The expression of IL-17 A and TNFα can be induced by Th17 cells-mediated inflammatory responses, which may result in bone and articular cartilage damage. However, Tregs exhibit anti-inflammatory activity by promoting the expression of IL-10 and TGF-β. The aberrant control of Treg/Th17 cells can lead to the inflammatory responses in RA (Fahey and Doyle, 2019).

The low-graded chronic inflammation in the synovium can induce the formation of pannus, which is granulation tissue with high vascularization. Pannus is often formed at the marginal sites of diarthrodial joints, facilitating to invade and impair the nearby cartilage and subchondral bone. The interaction between T cells and macrophages may induce complex immune responses, producing a large number of pro-inflammatory cytokines, such as IL-1, IL-6, and TNFα. These catabolic factors have been involved in the destruction of ECM and cartilage by activating MMPs or ADAMTSs (McInnes and O'Dell, 2010; Orr et al., 2017). The anti-inflammatory activity of genistein has been implicated in the potential therapeutic management of RA. In RA rats, genistein can significantly reduce collagen-induced inflammation, as indicated by lower ear thickness, decreased productions of TNFα, IL-6, adiponectin, and leptin in the serum, and improved joint destruction (Mohammad-Shahi et al., 2011) (Table 3).

It has been reported that genistein can significantly decrease the productions of IL-1β, IL-6, and IL-8 in TNFα-treated MH7A cells. Additionally, genistein inhibits TNFα-induced phosphorylation of AKT, IκBα, IKKα/β, and p65, blocks p65 nuclear translocation, and abolishes NF-κB transcriptional activity. Moreover, genistein exhibits anti-oxidative activity by decreasing the production of ROS and activates AMPK functions in TNFα-treated MH7A cells (Li et al., 2014) (Table 3). Another study reports that IL-1β, TNFα, and EGF have been shown to support the long-term inflammation, contributing to the proliferation and transformation of fibroblast-like synoviocytes (FLS) (Pap et al., 2005). These effects can be blocked by genistein through inactivating ERK1/2 signaling pathway. Further study indicates that genistein suppresses IL-1β- or TNFα-induced production of MMP-2 and MMP-9. Interestingly, EGF only increases the expression of MMP-9 and has no effects on MMP-2 expression. Genistein can also exhibit inhibitory activity against EGF-induced MMP-9 expression by inactivating JNK in RA synoviocytes (Zhang et al., 2012) (Table 3).

Angiogenesis has become an obvious characteristic of pannus formation, and the blood vessels development contributes to nutrition supply for synovial membrane proliferation and facilitates to transport the inflammatory cytokines and cells (Clavel et al., 2003) (Figure 4). As a result, angiogenesis plays a critical role in the progression of RA, and it can be a potential target for RA therapeutic management. It has been demonstrated that inhibition of angiogenesis may lead to amelioration of synovial inflammation and blockage of pannus formation, inhibiting RA pathological development (Szekanecz and Koch, 2009). In collagen-induced mice arthritis, genistein has been reported to suppress the expression of IL-6, IL-1β, and TNFα in the serum, decrease VEGF expression and angiogenesis in the synovial membranes, and alleviate the joint structure damage (Hu et al., 2016). IL-6 is known to trigger the expression of VEGF by stimulating JAK/STAT3 signaling pathway. Consistently, it has been demonstrated that genistein decreases the expression of IL-6, VEGF, and STAT3 in MH7A cells. In addition, genistein also suppresses the migration of vascular endothelial cells and the formation of tube in IL-6-stimulated EA.hy926 cells (Cheng et al., 2020) (Table 3).

The activity of Th1 cells is more associated with the innate immunity, eliminating the host pathogens. In contrast, Th2 cells activity is related to the activation of B lymphocytes and the production of antibody. A fine tune between Th1 and Th2 cell is dedicated, and disturbance of this relationship may lead to chronic pathological changes, such as allergy, auto-immune disorders, and cancers. It has been reported that genistein may direct the balance of Th1/Th2 towards the Th2 response by decreasing the ratio of IFNγ/IL-10 in stimulated murine splenocytes (Rachoń et al., 2006). Consistently, genistein may block the proliferation of splenocytes, decrease the production of IFNγ, and increase the expression of IL-4, maintaining the balance of Th1/Th2 activities in collagen-induced rat RA. T-bet and GATA-3 are the central mediator in the differentiation of Th1 and Th2 cells, respectively. Genistein has been shown the inhibitory activity against T-bet expression and stimulating activity on GATA-3 expression, shifting to Th2 response (Wang et al., 2008). In collagen-induced mice arthritis, genistein exhibits protective activity against osteoclasts functions by enhancing the glycosylation of IgG and subsequently ameliorating the inflammatory responses, such as NF-κB and NFATc1/c-Fos signaling pathways (Du et al., 2020) (Table 3).

Collectively, many factors, such as oxidative stress, inflammation, immune alternations, and angiogenesis have been demonstrated to facilitate the pathogenesis of RA. Hopefully, genistein can ameliorate the pathological changes induced by these detrimental stimuli. Many signaling pathways, such as NLRP3, TGF-β, NF-κB, AMPK, and JAK/STAT3, have been involved in the regulatory activity of genistein against RA pathological development.

### Genistein and intervertebral disc degeneration

**FIGURE 5:**
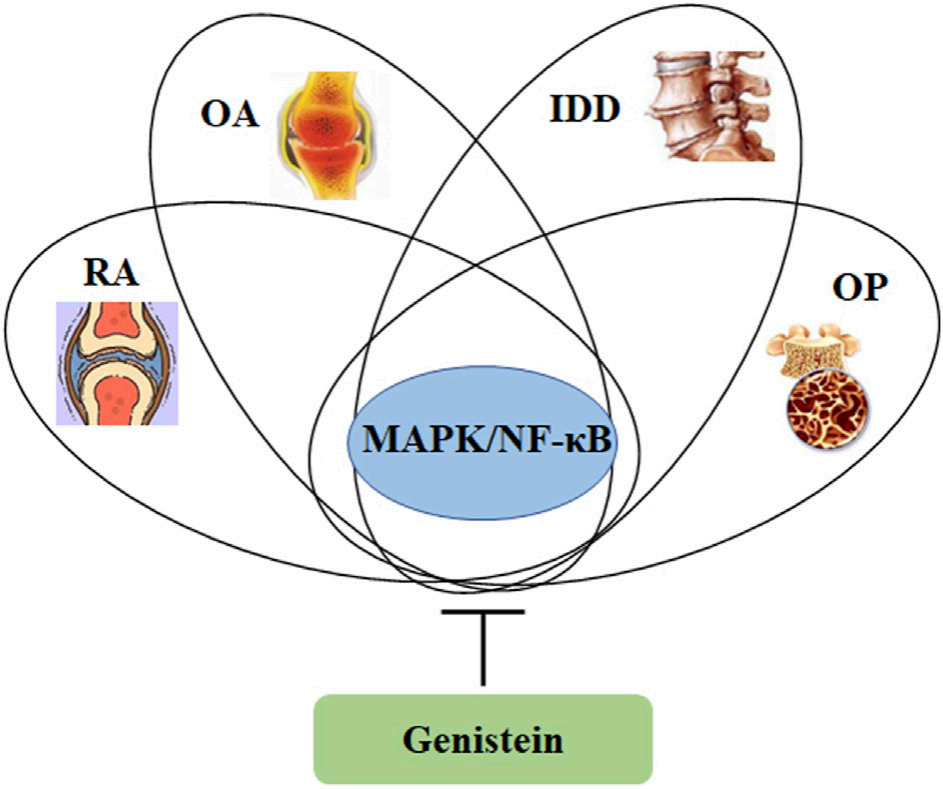
Genistein can inhibit the activity of MAPK/NF-κB signaling pathway, which is involved in the pathological development of osteoporosis, intervertebral disc degeneration, osteoarthritis, and rheumatoid arthritis.

Intervertebral disc degeneration (IDD) is a pathophysiological change in which the intervertebral disc (IVD) degenerates with aging, due to a comprehensive interaction between environment and genetic factor. Nowadays, many available therapeutic strategies are implicated in IDD treatment, which mainly involves operational therapy and non-operational treatment (drug treatment). However, each therapeutic strategy has its limitations (Sharifi et al., 2015). These might be associated with the lack of fully understanding of the potential molecular mechanisms of IDD development. The components of IVD include nucleus pulposus (NP, annulus fibrosus (AF), and cartilaginous endplates (CEP). Most of ECM is synthesized by NP cells. Maintenance of NP cell functions and ECM microenvironment from damage has been the potential target for IDD management. It has been comprehensively discussed that inflammation, oxidative stress, apoptosis, and mechanical overloading are associated with the pathological development of IDD. Many signaling cascades, such as MAPK, NF-κB, and NRF2/HO-1 signaling pathways, are orchestrated to mediate the pathophysiological actions in IVD cells (Costăchescu et al., 2022) (Figure 5).

Phytoestrogen has been found to protect IVD from degeneration by inhibiting the transcriptional expression of MMP-13 and ADAMTS-4, increasing the production of PG, and ameliorating cell apoptosis in bFGF- and IL-1-treated NP cells (Li et al., 2008). Recently, oxidative stress has been reported to exhibit a critical role in the pathogenesis of IDD (Jiang et al., 2018). Tert-butyl hydroperoxide (TBHP), a trigger of oxidative stress, has been reported to induce the degenerative changes in NP cells, as demonstrated by increased cell apoptosis and ECM degradation (Chen et al., 2018). Genistein has been shown anti-oxidative stress against TBHP-induced NP cells apoptosis and ECM degradation. Specifically, genistein at the dose of 100 μM reduces the expression of MMP-13 and ADAMTS-5, increases the productions of aggrecan and type-II collagen, and protects IVD tissue structure by stimulating the activity of NRF2/HO-1 signaling pathway (Wang et al., 2019) (Table 3). Inflammation responses also facilitate the pathological development of IDD. Mitogen-activated protein kinase (MAPK) signaling pathway (includes p38 MAPK, ERK1/2, and JNK) exhibits an essential role in signal cascades mediating inflammation. Activation of MAPK signaling is associated with increased production of IL-1β, IL-6, and TNFα. Up regulated expression of MAPK has been observed in NP cells, correlating with the development of IDD (Liu et al., 2016). This suggests that MAPK signaling pathway has been a potential target for therapeutic management of IDD. P38 MAPK is a protein tyrosine kinase (PTK), and genistein has been considered as an inhibitor of PTK. It has been demonstrated that genistein can effectively ameliorate the inflammatory responses (IL-1β, TNFα, and NF-κB) and delay the degeneration of IVD tissue (MMP-13, aggrecan, and collagen-II) by inhibiting the phosphorylation of p38 MAPK *in vivo* and *in vitro* (Ge et al., 2020) (Table 3).

Collectively, genistein shows anti-oxidative and anti-inflammatory activities against the development of IDD by mediating NRF2/HO-1 and MAPK/NF-κB signaling pathways.

### Clinical perspectives and limitations

In a randomized and double-blind trial for 24 months, genistein (54 mg/kg/day) exhibits beneficial effects on BMD of the spine and hip in osteopenic postmenopausal Caucasus women (Marini et al., 2007). Another one randomized double-blind placebo-controlled study shows that genistein exhibits positive effects on bone loss, suggesting stimulation of bone formation and suppression of bone resorption (Morabito et al., 2002). In patients with Sanfilippo disease, soybean isoflavone extract containing genistein (10 mg/kg/day for 12 months) has been demonstrated to decrease the urinary excretion of glycosaminoglycan and plasma heparan sulfate levels (de Ruijter et al., 2012). Many key findings of genistein on clinical studies for potentially therapeutic use have been summarized in anti-cancer, anti-diabetes, and neuroprotection (Sharifi-Rad et al., 2021). Although the protective effects of genistein in ameliorating bone loss during menopause have been well known, more relative clinical trials are still needed for further investigated.

Genistein has a low water solubility and poor bioavailability, which have become the obstacle for genistein in developing as a potential candidate in functional foods or health-benefiting medicine. In addition, genistein can be metabolized in a short time after oral administration, leading to aberrant absorption (Jung et al., 2020). Hopefully, many strategies have been developed for improvement of water solubility, bioavailability, and structural stability. For example, a set of nanoparticles, consisting of zein and zein/carboxymethyl chitosan (CMCS), has been developed to encapsulate genistein, and they have been demonstrated to be effectively compromising these shortcomings and serve for better food or pharmaceutical applications (Xiao et al., 2020). Another controversial issue might be associated with the estrogenic and goitrogenic activities of genistein, which can lead to potential adverse effects after consumption (Messina, 2016). It has been previously reported that genistein exhibits as an ER agonist or antagonist and is considered as a selective estrogen receptor modulator (higher activity in binding to ERβ than to ERα) (Setchell, 2001). However, no adverse effects on male fertility (Messina, 2010) and reduced risk of breast cancer (Maskarinec et al., 2017) are observed by excess consumption of soy or genistein. Recently, the impact of genistein on thyroid functions has been intensively discussed due to being as an inhibitor of thyroid peroxidase (TPO) *in vitro* (Hüser et al., 2018). In healthy adults, the negative effects of genistein on thyroid functions do not appear (Messina and Redmond, 2006). Many experimental results from *in vitro* or animal studies cannot be directly extrapolated to humans.

## Conclusion

Genistein, the richest isoflavone from soybean, has been demonstrated various biological effects, including anti-inflammation, anti-oxidation, and bone protection. The estrogen-like activity of genistein potentially facilitates its applications in therapeutic management of osteoporosis and osteoarthritis. Many other signaling pathways, such as MAPK, NF-κB, NO/cGMP, RANKL, NRF2/HO-1, and JAK/STAT3, have been involved in the network of regulation orchestrated by genistein in protecting against bone and cartilage diseases (Figure 5). Particularly, genistein can suppress angiogenesis to inhibit pannus formation, which is a classical feature of RA. Furthermore, genistein also modulates immune system for maintaining the balance of Th1/Th2 and shifting to Th2 responses (Wang et al., 2008). These data demonstrate that genistein can be a promising potential candidate for therapeutic use in bone and cartilage diseases. However, the molecular actions of genistein *in vivo* and *in vitro* are not recognized very well yet, and more efforts are still needed in the understanding of its pharmacological parameters and toxicity in the laboratory and clinical trials. In addition, the health benefiting effects of genistein in human study are still needed for further investigation. Collectively, genistein has been demonstrated the beneficial effects on bone and cartilage diseases.
